# Numerical Investigation of FRCM-Strengthened Corroded RC Beams under Cathodic Protection

**DOI:** 10.3390/ma15155334

**Published:** 2022-08-03

**Authors:** Kurdo Abdulla, Xiaoming Zhu, Meini Su

**Affiliations:** 1 School of Engineering, Faculty of Engineering and Creative Technologies, University of Bolton, Deane Road, Bolton BL3 5AB, UK; k.abdulla@bolton.ac.uk; 2 Department of Mechanical, Aerospace and Civil Engineering, University of Manchester, Manchester M13 9PL, UK; xiaoming.zhu-2@manchester.ac.uk

**Keywords:** composite materials, corrosion, FRCM, ICCP, numerical modelling, simply supported beam, structural strengthening

## Abstract

Fibric reinforced cementitious matrix (FRCM) composites have been used to improve the mechanical performance of reinforced concrete beams subjected to degradation in the past decades. Recently, dual-functional carbon fibres have been explored to provide both structural strengthening to RC beams and cathodic protection to reinforcement bars. This paper investigates the loading responses and structural behaviour of RC beams subjected to different levels of corrosion, protected by impressed current cathodic protection and structurally strengthened by external bonded FRCM. A numerical model is developed for the corroded RC beams under impressed current cathodic protection and structural strengthening by the FRCM composite. Upon validation against experimental results collected from the literature, the finite element model is then used for parametric study. A number of numerical results are generated to analyse the effects of key parameters, including the corrosion rate, degradation level of interfacial bonding properties due to anode acidification, and end anchorage, followed by detailed discussions. It is found that the significance of the corrosion of steel reinforcement bars significantly affects the load-carrying capacity of the beams. Increasing the corrosion rate from 0 to 40% reduces the load-carrying capacity of un-strengthened beams to 45% of the original capacity. Therefore, the cathodic protection provided by the C-FRCM plate is important to the reinforcement bars as it can avoid the cross-section area reduction of reinforcement bars and, thus, the main loading capacities of the beams. In this study, the degradation of the bonding properties at the interface of carbon fibre and the cementitious matrix due to anode acidification during impressed current cathodic protection is also considered. It is found that the bond strength of the C-FRCM plate has a slight effect on the load-carrying capacity of the beam. In addition, the application of end anchorage can significantly enhance both the load-carrying capacity and ductility of the beams. The rates of enhancement, if compared to the beams with no end anchorage, can reach up to 60%.

## 1. Introduction

Chloride-induced reinforcement bar (rebar) corrosion is one of the major forms of environmental attack on reinforced concrete (RC) structures [1,2], and inevitably causes a reduction in the strength, serviceability and aesthetics of the contaminated structures. The main solutions to this problem can be categorised as: (a) use of advanced materials, e.g., fabric-reinforced polymers (FRP) and fabric-reinforced cementitious composites, for external RC structure repair [3,4]; (b) the use of electrochemical techniques to protect reinforcement bars and prevent further corrosion [5,6]; and (c) the employment of nanotechnology for structural monitoring, such as real-time sensors [7,8]. Researchers also started to explore the possibility of combining the merits of different techniques. Lambert et al. [5], Gadve et al. [9] and Bahekar et al. [10] installed FRP using epoxy resin to the soffit of corrosion-damaged beams or concrete cylinders, and, meanwhile, applied an impressed current to protect the steel reinforcement bars. It was found that the corrosion of reinforcements was prevented due to the cathodic protection; meanwhile, the flexural loading capacities of these beams were also significantly improved in comparison to control beams. However, in these cases, the epoxy resin agent is not applicable in low temperatures and moist environments due to an aging problem.

Recently, fabric reinforced cementitious matrix (FRCM) composites have attracted attention as a feasible alternative for combining impressed current cathodic protection and structural strengthening (ICCP-SS) [11]. Zhu et al. [12] and Su et al. [13] firstly proposed to use FRCM as the dual functional material ICCP-SS intervention for RC structural members subjected to corrosion. In this intervention method, the carbon-fibre meshes embedded in FRCM functions as an anode material for ICCP and as an external strengthening material for SS [14]. A number of experimental programmes have been carried out to demonstrate the effectiveness of the ICCP-SS dual-intervention system on corroded structural members, including short columns under static loads [15] and under cyclic loads [16], simply supported beams [17,18] and continuous beams [19,20] under static loads, as well as simply supported beams [21] and continuous beams [22,23] under cyclic loads. Specifically, results from research [15] reveal that the ultimate compressive loads of columns intervened upon by the ICCP-SS technique could be improved by up to 50.4% compared to control columns. In terms of ICCP-SS application on flexural members, Su et al. [17] bonded a carbon FRCM (C-FRCM) plate to the soffit of simple support beams for both ICCP applying and external strengthening, and results indicated a 19.2% to 41.8% increase in flexural bearing capacity. A long-term experimental program was later conducted by Zhu et al. [18] to investigate the optimal range of charge density and protection duration for mechanical-behaviour enhancement. A similar improvement in the flexural performance of RC continuous beams with ICCP-SS was also reported by Su et al. [19] and Feng et al. [20]. In all of the abovementioned studies, electrochemical signals were measured and compared against standard criteria to ensure the effective protection of reinforcement bars. Except for the static loading condition, Su et al. [22] and Wei [24] proved the flexural bearing capacity of concrete structures are substantially improved after the application of ICCP-SS for beams subjected to cyclic loads. These experimental programmes proved that the ICCP-SS intervention system can not only effectively prevent steel reinforcement corrosion, but also restore the loading capacities of degraded structural members. The ICCP-SS system has now been applied in a new tunnel project in Guangdong province, China [25].

When using FRCM to strengthen RC beams, it should be noted that the failure modes of FRCM strengthened beams are different from traditional FRP-epoxy resin repaired beams. It is characterized by fabric slippage or rupture failure under lower-amount-of-fabric-ratio scenarios, while an FRCM-composite debonding failure or concrete-cover peeling-off failure occur under higher-amount-of-fabric-mesh scenarios. Furthermore, an impressed current has been found to have potential adverse effects on the bonding interface, where the bonding between carbon fibres and matrix (i.e., anode in ICCP system) could cause calcium leaching due to anodic polarization [26,27]. The acidification in anodes, which is caused by a large current density or long duration, makes the interfacial bonding properties crucial to the structural strengthening design [28]. Such interfacial mechanism degradation largely compromises the performance of the ICCP-SS intervention system and structure service life [29,30]. However, there is no study to show how the corroded beams repaired by ICCP-SS intervention will respond to different levels of interface degradation in a long-term timeframe. Although the positive effects of the ICCP-SS intervention method on corroded beams in a short-term timeframe have been validated by experiments, its long-term effect is still unclear, especially when severe interface degradation occurs. It is difficult and time-consuming to carry out this kind of tests, since the degradation of carbon fibre and the matrix interface would only occur after years of applying ICCP. Therefore, numerical modelling is considered a more reasonable approach in this case. Currently, there is no numerical investigation into the mechanical behaviour of corrosion-damaged structural members enhanced by the ICCP-SS intervention system with an FRCM composite plate. Only a few have shed light on the modelling of concrete structural strengthening with FRCM composites [31,32,33,34], but without considering the involvement of the ICCP technique. Overall, numerical investigation is needed to help better understand how interfacial degradation plays a role in the response of structural members intervened by both SS and ICCP over a long-term timeframe.

This paper will, firstly, present a finite element (FE) model of reinforced concrete beams strengthened by FRCM composites. The model will then be validated against experimental results collected from the literature. Upon validation, a parametric study will be carried out to explore the effects of key parameters that might affect the behaviour of corroded RC beams with ICCP-SS in practical cases. A detailed discussion will be carried out based on the newly generated numerical data. Key parameters considered in this study include the initial corrosion rate of reinforcement bars, the degradation level of the carbon fibre and matrix interface due to cathodic protection, as well as the end-anchorage condition of carbon-fibre meshes. Finally, the flexural capacities of the simulated beams will also be compared to the strengths predicted by the international design standards to show the applicability of the design standards to the corroded RC beams with long-term ICCP-SS protection.

## 2. Experimental Results from the Literature

A total of 11 corroded RC beams subjected to four-point bending is reported by Su et al. [6], where specimens were repaired by either ICCP, SS or ICCP-SS techniques. Except for reference beams, all the specimens were exposed to an accelerated corrosion process after the curing period. The accelerated corrosion was achieved by adding an amount of NaCl, 3% of the weight of cement, during the concrete mixture. In specimens strengthened with the ICCP technique, after an accelerated corrosion period, constant current densities were directly applied to the reinforcement. Meanwhile, for specimens with the SS and ICCP-SS technique, carbon FRCM composite plates were bonded onto the full soffit side of the beam first. Two kinds of imposed current, 26 mA/m^2^ and 80 mA/m^2^, were applied to the ICCP-SS specimens, whereas, for SS specimens, it was not implemented. The total length of the beam was 1300 mm and the cross-section dimension was 150 mm (height) × 100 mm (width). Two 10 mm (in diameter) longitudinal reinforcement bars were located at the bottom of beams with concrete cover of 30 mm. During a four-point bending test, two supports were installed 50 mm away from the beam ends and a spreader beam was employed to form a pure bending length of 400 mm. The details of the beam dimensions, reinforcing bars and boundary conditions, plus the experimental results, including the failure modes and bending capacities of specimens, are detailed in literature [17].

## 3. Numerical Modelling and Validation

Finite element (FE) analysis is conducted by employing a commercial FE software Abaqus CAE 2021. The numerical modelling developed in this study is performed with reference to the aforementioned beams.

The FE models of the ICCP-SS strengthened RC beams were generated using a combination of constitutive models to capture both the linear and non-linear behaviour of the beams. The concrete for the beam and the cementitious layer were assumed to be homogenous materials so as to simplify the simulations and reduce computational time. These parts were modelled using 3D linear brick elements with eight nodes and reduced integration and hour-glass control (type C3D8R) [35]. The elastic moduli of the concrete and cementitious materials were defined to capture their linear behaviour, while the concrete damaged plasticity model was employed to capture their non-linear behaviour. Tensile damage parameters were also defined for the concrete part to obtain possible tensile cracks from the model output. The elastic moduli values and other parameters used to define both the concrete and cementitious materials are summarised in Table 1. It is also worth mentioning that the part of the cementitious layer, which is used to cover the FRP mesh in the original experiments and does not have significant structural contributions, was not included in the FE models to simplify the analyses.

The steel bars were modelled using truss elements with two nodes (type T3D2); elastic and plastic models were employed to capture both linear and non-linear responses of the bars. The data used to define the mechanical properties of the steel bars are defined in Table 2; the data was obtained based on the experimental tests conducted by the authors. In order to account for the reduction in the strength of steel bars as a result of corrosion (as reported in the original experiments [6]), the effective cross-sectional area of the reinforcement bars was reduced in the FE models. Different reduction rates in the cross-sectional area were adopted in the FE models to account for the effects of ICCP observed in the original experiments. The FRP mesh, which is used as the external strengthening solution, was simulated using shell elements with reduced integration (type S4R). 

A surface-based cohesive behaviour model was employed to define the mechanical interaction at the surfaces between the cementitious layer and concrete beam and between the FRP mesh and the cementitious layer. The linear response between the surfaces was simulated based on a linear traction separation relationship, where the components of the stiffness matrix were defined, i.e., stiffness at the interfaces in normal and shear directions (see Table 3). The possible damage initiation between the surfaces were included in the simulations by defining quadratic stress criterion, in which tensile and shear bond strength at the interfaces were defined, as shown in Table 3. The propagation of damage at the contacting surfaces was captured via the definition of the fracture energy with linear softening. Hard contact behaviour was also defined between the surfaces to prevent the penetration and between the contacting surfaces. In addition, the contacts between surfaces were defined through a node-to-surface discretisation method with a finite sliding formulation.

**Figure 1:**
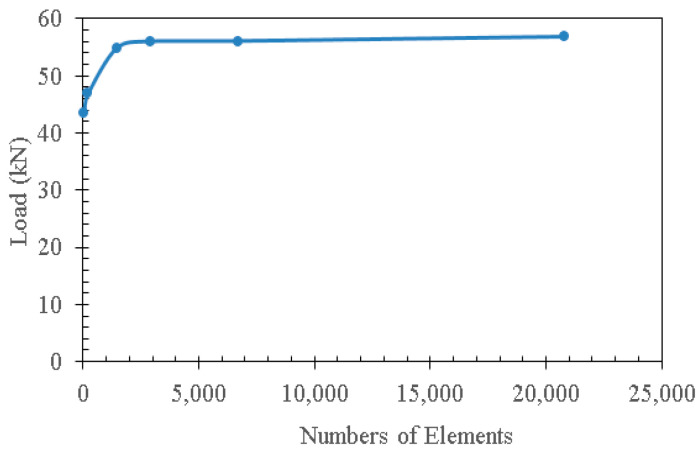
Comparison of the load-bearing capacity obtained from different mesh sizes.

The boundary conditions were applied via the definition of rigid bodies to simulate the steel plates used in the experiments. At the supports, the horizontal and vertical transitions were restrained while the rotation was released to simulate the simply supported condition. At the loading plate, the vertical force was applied under displacement control. The mesh size sensitivity was analysed by modelling the four-point bending tests on specimen C0-Pt-D20 using a series of models with six different mesh sizes (see Table 4). The effect of mesh size on the load-bearing capacity is plotted in Figure 1. Finally, a mesh size of 20 mm (Model 4 in Table 4) was chosen for this study by considering both the deviation in load and the computation cost. A general non-linear static procedure was adopted to analyse the models. 

**Figure 2:**
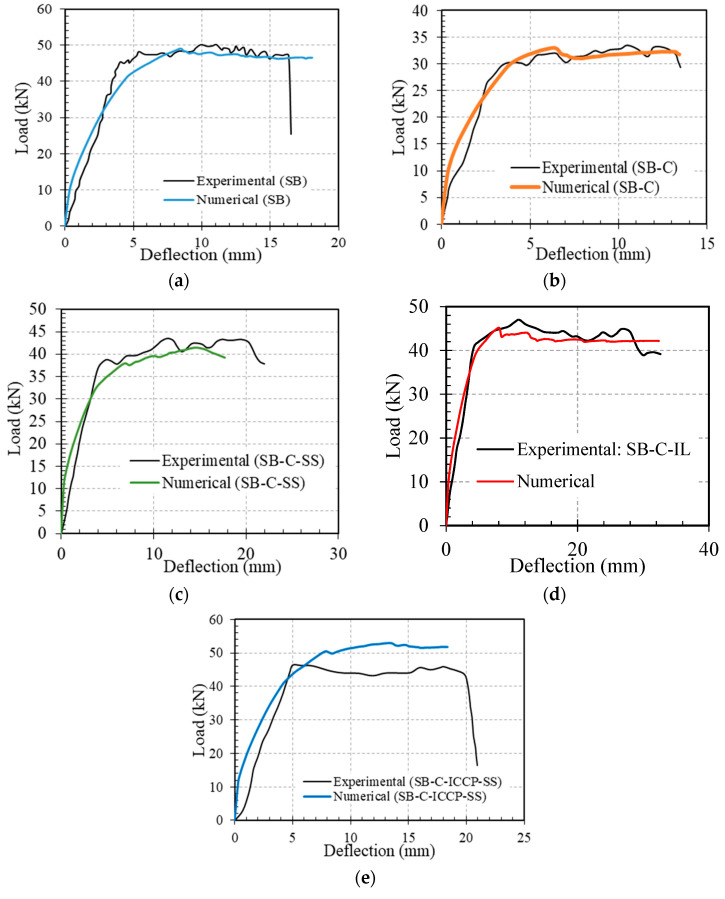
Comparison between experimental and numerical results: (**a**) the reference beam, (**b**) the beam with corroded steel bars, (**c**) the beam strengthened by a C-FRCM layer only, (**d**) the beam protected by ICCP only, and (**e**) the beam strengthened by ICCP-SS.

**Figure 3:**
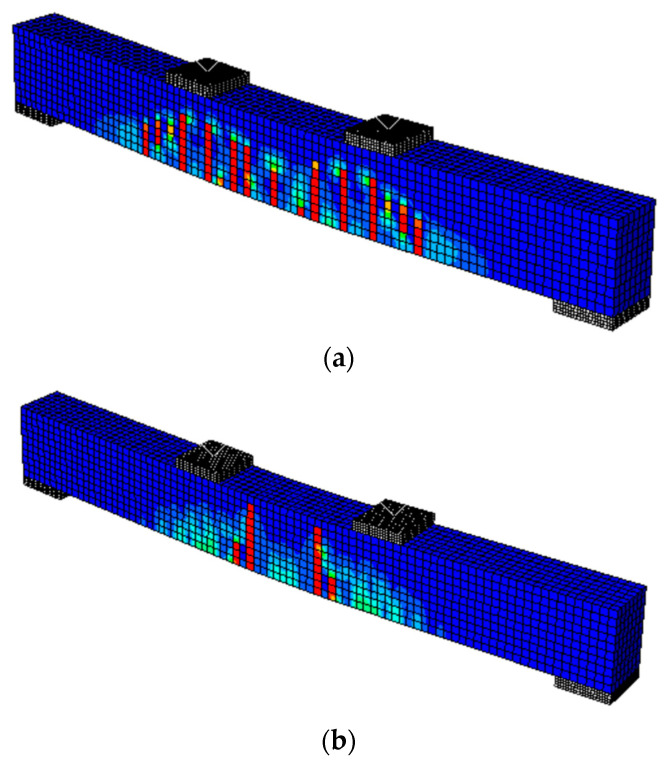
Flexural cracking of concrete beams, (**a**) the reference beam, (**b**) the beam with corroded steel bars.

**Figure 4:**
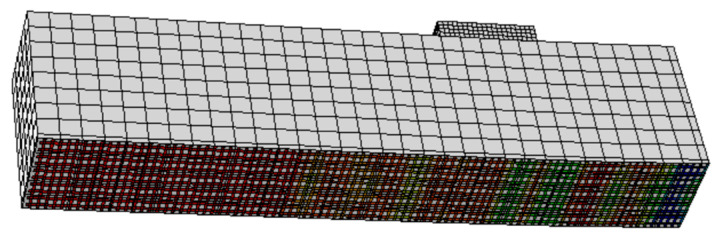
Shear slippage of C-FRM layer in the beam strengthened by an ICCP and C-FRM layer.

In the validation studies, five models were developed to simulate five different cases, namely, the reference beam (SB), beam with corroded steel bars (SB-C), beam with corroded steel bars strengthened by CFRM layer (SB-C-SS), beam with corroded steel bars strengthened by ICCP (SB-C-ICCP), beam with corroded steel bars strengthened by ICCP and CFRM (SB-C- ICCP-SS). The numerical results showed a good agreement with the experimental tests in terms of linear and non-linear load displacement responses, as shown in Figure 2. For the beam strengthened by ICCP-SS, there is a difference between the FE and experimental results (see Figure 2e); this difference is due to the limitation of simulating the actual bond strength and a lack of mechanical-properties data to represent the experimental bond strength between the FRCM layer and concrete beam. In addition, the failure modes captured by FE models were consistent with the experiments. For example, in the reference beam, the failure mode in the FE model and experiment was characterised by the formation of flexural cracks in the concrete; Figure 3 shows the locations of tensile damage, i.e., flexural cracks. Similarly, the failure modes of the beam with corroded steel bars were due to the formation of flexural cracks in both numerical analysis and experiments. The failure modes of the beams strengthened by a C-FRM layer were characterised by the slippage of the CFRM layer, cracking of concrete and yielding of rebars. Figure 4 shows the slippage of the CFRM layer for the beam strengthened by the C-FRM layer.

## 4. Parametric Studies

After the validation of the FE models, a series of parametric studies was carried out to investigate the effects of different key parameters on the structural response of the concrete beams to obtain detailed understandings of the ICCP-SS intervention system. Table 5 summarises the results generated from the parametric studies.

The parameters that are examined in this study include the initial corrosion rate of the steel reinforcement bars, degradation in the mechanical properties of joint interfaces and effects of the end anchorage of the FRCM strengthening layer on the structural performance of the beams. In total, 16 models were analysed. The details for the labelling and parameters associated with parametric studies are: the first part of the name indicates the corrosion rate of the reinforcement bars, the second part of the name shows whether the beam was under ICCP-SS protection, and the third part presents the degradation level of the anode interface after long-term cathodic protection. If end mechanical anchorage is provided for the FRCM plate, a letter “Y” is added to the name of the specimen. For example, a beam model with 0% corrosion in the reinforcement and 30% degradation in interface with an end anchorage of FRP mesh is denoted as C0-Pt-D30-Y. For 10% degradation rates for corrosion and interface properties, the effective reinforcement area is assumed to be 90% of the original area and bond strength at the interfaces to be 90% of the original strength.

## 5. Discussion

### 5.1. Effects of Degradation of Fibre/Cement Interface

**Figure 5:**
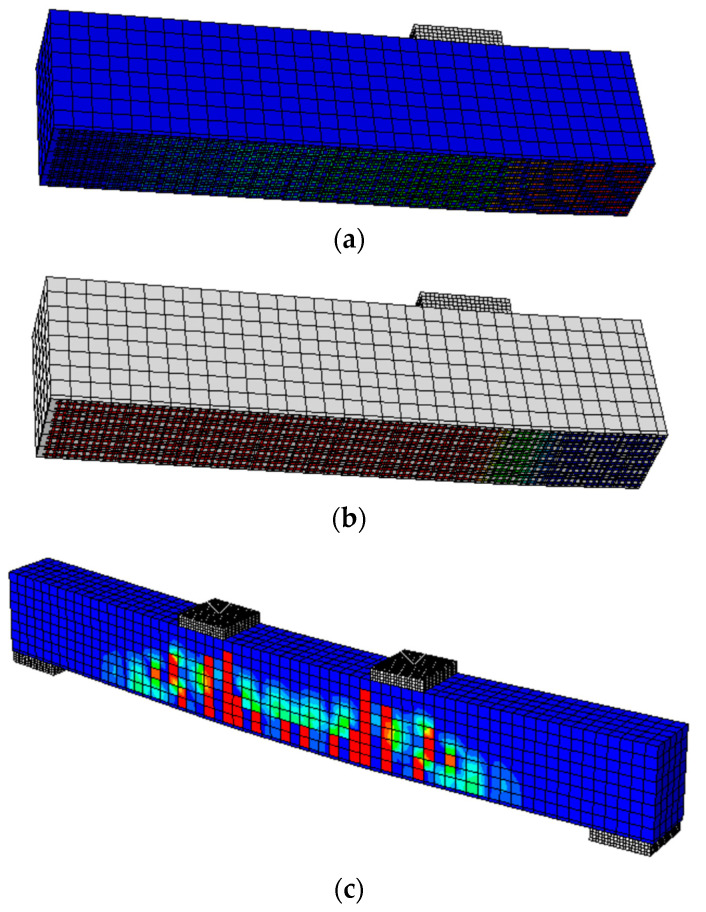
Typical failure mechanisms of beams with different CF/cementitious matrix bond interface strength (**a**) stress state in carbon fibre mesh just before the slippage occurs (MPa), (**b**) the slippage of the CF mesh (mm), (**c**) the flexural cracks in the concrete beam.

The degradation in the bond strength between the carbon-fibre mesh and cementitious matrix does not have significant effects on the ultimate load-carrying capacity of the beams. However, the ductility of the beams with a higher bond interface was relatively higher; this is referred to as the contribution of the bond interface, to absorb more energy and consequently provide higher deformation capacity. The effect of interface bond strength is not significant, because of the difference between the stiffness of the cementitious matrix and carbon-fibre mesh; a high bond strength and with no slippage cannot be achieved. In cases when a higher bond strength was assumed, the slippage still occurs prior to reaching the ultimate strength of the carbon-fibre mesh, see Figure 5a. All the beams with relatively different interface strength had the same failure mechanisms, which starts with slippage of the carbon fibre mesh layer, see Figure 5b, followed by the flexural cracks in concrete, as in Figure 5c. However, the C-FRCM layer contributes to protecting the beam and preventing the corrosion of the steel bars. In the cases where the beams are exposed to an extreme environment for a long time, if a beam has no protection and serious corrosion occurs in the reinforcement bar (such as with specimen C40), its capacity will be less than half of the reference beam (C0); however, if the beam is protected by ICCP-SS at the beginning, even after a long time and degradation occurring at the C-FRCM interface (such as specimen C0-Pt-D40), its capacity can still satisfy the design requirement (i.e., it will be higher than the capacity of beam at initial status (i.e., C0)). Therefore, it can be seen that the application of ICCP-SS intervention is efficient at maintaining the performance of RC structures.

### 5.2. Effects of Mechanical Anchorage

**Figure 6:**
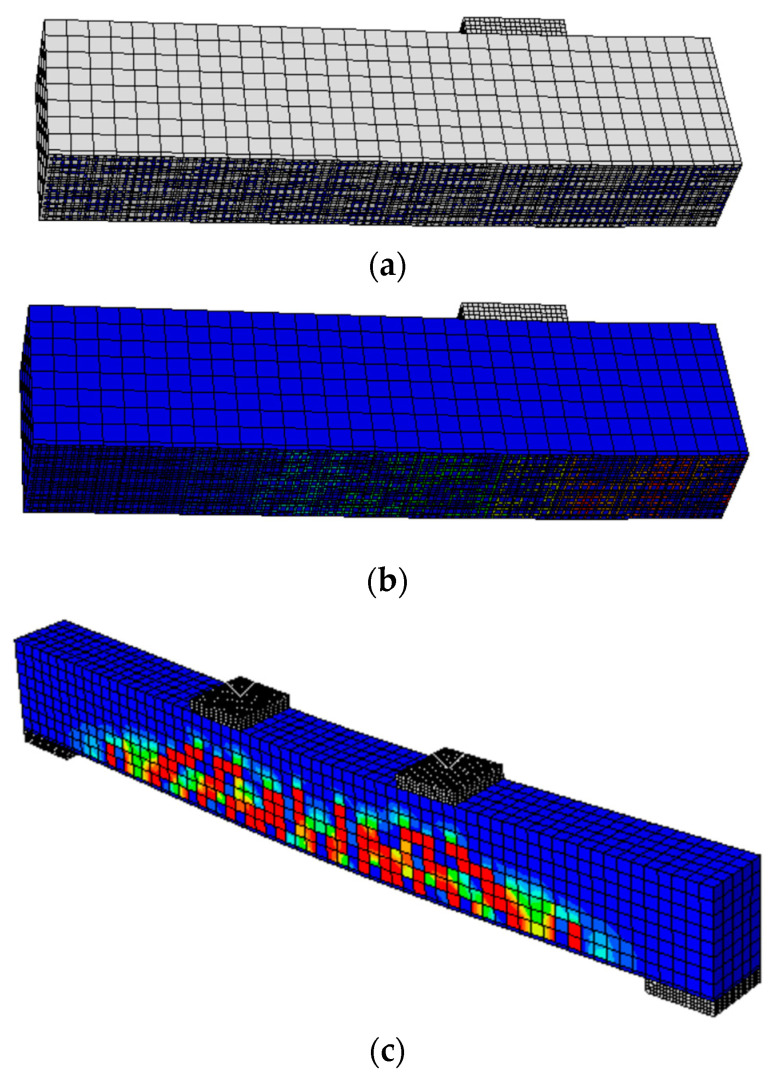
Typical failure mechanisms of beams with mechanical end anchorage: (**a**) zero slippage of CF mesh, (**b**) stress distribution in the CF mesh layer (MPa), (**c**) the flexural cracks distributed in the concrete beam.

The results show that introducing the end anchorage to the C-FRM layer significantly improves the load carrying capacity of the concrete beams. For the beams under the same conditions, applying the end anchorage increases the load carrying capacity of the beams by 20%. The end anchorage increased the load carrying capacity of the C0-Pt-D30 beam from 55.53 kN to 64.15 kN. Due to the end anchorage, the C-FRPM layers deform and stretch with the deformation of the concrete beam and contribute to taking stress, regardless of the bond strength degree. End anchorages prevent the slippage of the C-FRCM layer, as the beam deforms even when the interface bond is relatively weak, as shown in Figure 6a. As a result, the carbon-fibre mesh can contribute most of its strength to take stresses while the beam deforms, as shown in Figure 6b. The end anchorage also improves the deformation capacity of the beam by a factor of 1.6, as it can be noted from the results that the ultimate displacement for the beams with end anchorages is at least 1.6 times more than the displacement of the counterpart beams with no end anchorages. The failure mode of the beams with end anchorages is due to the formation of flexural cracks in the beam; as the beam deforms the carbon mesh continues to take stresses and absorbs more energy, then the stresses are re-distributed to the undamaged regions of the beam, which causes even distribution of the flexural cracks within the beam, as shown in Figure 6c. Consequently, the beam fails because of the failure of the carbon mesh and/or failure of the concrete, depending on the relative strength in between the carbon-fibre mesh and concrete. Therefore, it is suggested to provide the end anchorage by mechanical bonding configuration to the structural members in the practical applications.

### 5.3. Effects of Corrosion

The corrosion rate of steel bars has a significant effect on the load-carrying capacity of the beams. When the corrosion rates in the beam without C-FRCM increases from 0 to 40%, the load-carrying capacity drops by 55% (drops from 48.96 kN to 21.38 kN). This significant decrease in the load-carrying capacities of the beams will make them unsuitable for service, lose structural integrity and, finally, lead to safety concerns in real infrastructures. For the same corrosion rates with the presence of an C-FRCM layer, the drop in load-carrying capacity is not that sharp. In the C-FRCM strengthened beams, when the corrosion rate increases from 0 to 40%, the drop in load-carrying capacity is about 40%. This is due to the fact that the C-FRCM continues to take stresses after the steel reinforcement degrades and loses strength. The C-FRCM also improves the deformation capacity and provides more ductility to the beams. As the beams deforms, the carbon-fibre mesh layer dissipates energy and re-distributes the stresses in the beam even in the cases when the steel bars are excessively corroded. The presence of FRCM is critical in the beams with corroded steel bars to prevent the beams from brittle failure.

## 6. Comparison with Design Standards

The load-bearing capacity $F_{u}$ obtained from the FE analyses were also compared with the nominal design strength $F_{ACI318}$ predicted by the code ACI318 [37] for un-strengthened beams, the nominal design strength $F_{ACI549}$ and $F_{ACI440}$ by ACI 549.4R-13 [38] and ACI 440.2R-17 [39], respectively, for beams strengthened with FRCM composite. The comparison results are shown in Table 5.

In code ACI318 [37], the design bending moment capacity $M_{d}$ is composed by the resistance of steel reinforcement $M_{ns}$, while in the codes ACI 549.4R-13 [38] and ACI 440.2R-17 [39], the design bending moment capacity consists of two parts, the flexural strengths contributed by FRCM composite $M_{nf}$ and by steel reinforcement $M_{ns}$, respectively, as shown in Equation (1).

(1) $$M_{d}=\phi (M_{nf}+M_{ns})$$

where ϕ is a strength reduction factor, following the determination method of Equation (2) in the three guidelines.

(2) $$\phi =\left\{ \begin{array}{l} 0.9,\varepsilon_{sn}\geq 0.005\\ 0.65+\frac{0.25(\varepsilon_{sn}-\varepsilon_{sy})}{0.005-\varepsilon_{sy}}\\ 0.65,otherwise \end{array}\right.,\varepsilon_{sy}<\varepsilon_{sn}<0.005$$

where $\varepsilon_{sy}$ is the steel tensile yield strain, and $\varepsilon_{sn}$ is the final steel strain after n iterations for determination of neutral axis depth.

Note that ACI 549.4R-13 [38] is the only accessible guideline for structural strengthening design with FRCM systems. The key of this design approach is that an effective tensile strain $\varepsilon_{ACI549\_FRCM}$ is defined for the prediction of the flexural strength of a beam externally bonded by FRCM, which is the minimum between the design value of FRCM ultimate tensile strain $\varepsilon_{FRCMd}$ and a constant 0.012, as shown in Equation (3).

(3) $$\varepsilon_{ACI549\_FRCM}=\min(\varepsilon_{FRCMd},0.012)$$

Accordingly, the effective tensile stress that an FRCM composite can provide for structural strengthening is calculated following Equation (4).

(4) $$f_{ACI549\_FRCM}=\varepsilon_{ACI549\_FRCM}E_{FRCM}$$

The values for $\varepsilon_{FRCMd}$ (0.0104) in Equation (3) and $E_{FRCM}$ (195 Gpa) in Equation (4) are extracted from the results of a uniaxial tensile test on FRCM composite plates performed in the literature [17] in accordance with the rules in ACI434-16 [40].

ACI 440.2R-17 [39] is a design guideline for structures strengthened by the FRP system. The effective strain $\varepsilon_{ACI440\_}{}_{FRCM}$ is regulated between the minimum of a strain $\varepsilon_{fd}$ at which debonding may occur and 90% of the ultimate tensile strain of dry fibre $\varepsilon_{fu}$, see Equations (5) and (6). Then, the effective tensile stress of FRCM can be derived by Equation (7).

(5) $$\varepsilon_{ACI440\_}{}_{FRCM}=\min(\varepsilon_{fd},0.9\varepsilon_{fu})$$

(6) $$\varepsilon_{fd}=0.41\sqrt{\frac{f_{c}^{\prime }}{E_{f}t_{f}}}$$

(7) $$f_{ACI440\_FRCM}=\varepsilon_{ACI440\_FRCM}E_{f}$$

where $f_{c}^{\prime }$ is the specified compressive strength of concrete, $E_{f}$ and $t_{f}$ is the modulus of elasticity and the nominal thickness of fibre reinforcement, respectively.

According to Table 5, for corroded beams without external strengthening of FRCM composite plate, the $F_{u}/F_{ACI318}$ ratio of the load-bearing capacity from modelling output and the results calculated by ACI 318 is around 1.34. It shows that the flexural-strength prediction approaches in standard ACI 318 is conservative for beam design. For corroded beams strengthened with FRCM composite plates and treated with the ICCP technique, the ratio for $F_{u}/F_{ACI549}$ and $F_{u}/F_{ACI440}$ is around 1.31 and 1.36, respectively. This generally indicates that both the standard ACI 549.4R-13 [38] and ACI 440.2R-17 [39] underestimated the loading-bearing capacity of beams. That is due to the effective strain limited in the two codes to avoid an intermediate cement crack-induced fibre debonding failure mode, which conservatively estimates the deflection capacity of fibre reinforcements. However, as mentioned earlier, fibre reinforcements have experienced debonding from cementitious matrix and even experienced different degrees of slippage at beam failure. Take beam C0-Pt-D10 as an example, the effective strain of the FRCM composite is predicted as 0.0104 and 0.0087 by equations codified in ACI 549.4R-13 [38] and ACI 440.2R-17 [39], respectively, while the measured fibre tensile strain at beam failure is 0.0124; in addition, the strain at ultimate of dry fibre is 0.0158 based on the tensile property of fibre provided in the literature [17]. For beams with an anchorage end (C0-Pt-D30-Y and C0-Pt-D40-Y), the theoretical results are much more conservative, leading to a greater ratio of $F_{u}/F_{ACI549}$ and $F_{u}/F_{ACI440}$ at around 1.40 and 1.47. This is because the mechanical anchorage at the ends enhances the interfacial behaviour between the fibre mesh and cement paste, so that the debonding and slippage failure can be largely avoided. In this case, the strain that the fibre mesh can reach is actually near the ultimate tensile strain of dry fibre. The full contribution of fibre largely improves the overall bearing capacity of the beams.

## 7. Conclusions

This paper presented a numerical study to investigate the structural behaviour of corroded reinforced concrete beams strengthened by fibre-reinforced cementitious matrix (FRCM) composites. The FRCM composite is used as a dual functional material to provide both structural strengthening for the beam as well as cathodic protection for the reinforcement bars. The newly developed model was validated against results obtained from tested beams. Afterwards, a parametric study was conducted to investigate the effects of governing parameters on the improvement in the mechanical performance of the degraded reinforced concrete beams. Key parameters, including the corrosion rate of reinforcement bars, the bonding behaviour between the carbon-fibre meshes and cementitious material and the end anchorage of carbon fibre meshes, were considered in this study. Results show that increasing the corrosion in the steel bars significantly reduces the load-carrying capacity of the beams. For severe corrosion cases, the drop in the load-carrying capacity can reach 55%. In this case, impressed current cathodic protection is important to protect the steel-reinforcement bars and thus to maintain the original strength of the beams when subjected to extreme environments. The bond strength between the carbon fibre and cement matrix has a slight effect on the loading capacities of the beams, but a more prolonged influence on the durability of the beams. Application of the end anchorage is effective to increase both the load-carrying capacity and ductility of the beams. End anchorage prevents the slippage at the interface between the beam and carbon-fibre-reinforced cementitious layer; consequently, the carbon-fibre mesh contributes to taking extra stresses compared to the cases with no end anchorage.

The limitations of the developed FE model can be summarised as the difficulty to define the bond strength between the FRCM layer and concrete bond due to the lack of realistic bond strength data. Another limitation is that when the end anchorage is defined, the model assumes there is no slippage in between in the FRCM layer and the concrete; however, this is not perfectly achievable in practice. Finally, the FE models did not actually simulate the corrosion of steel bars; the corrosion was assumed by a reduction in the original cross-sectional area of the steel bars.

The current study investigated the effects of steel-bar corrosion, mechanical end anchorage and interface degradation between fibre and cement on the structural behaviour of concrete beams strengthened by ICCP and an FRCM layer applied to the bottom face of the beam. Further studies can be conducted to study the effects of the configurations of the FRCM strengthening layer on the behaviour of the beam; the FRCM layer can be applied to the sides as well as the bottom face or used to wrap the concrete beam.

## Figures and Tables

**Table 1:** Properties of concrete and cementitious materials [17].

Parameters	Concrete	Cementitious Layer
Elastic modulus (MPa)	34,215	76,000
Poisson’s ratio	0.2	0.2
Tensile strength (MPa)	3	2.06
Compressive strength (MPa)	53	20.69

**Table 2:** Mechanical properties for steel reinforcement bars.

Elastic modulus (MPa)	190,000
Poisson’s ratio	0.3
Yield stress (MPa)	541.69
Strain at yield point	0.00157
Stress at failure (MPa)	642.84
Strain at failure	0.07887

**Table 3:** Mechanical properties at the joint interfaces.

Parameters	Concrete–Cementitious Layer Interface	Cementitious Layer–FRP Mesh Interface
Stiffness in normal direction (N/mm^3^)	15,000 (empirical)	20 [29]
Stiffness in shear directions (N/mm^3^)	10,000 (empirical)	15 [29]
Strength in normal direction (N/mm^2^)	2 [36]	0.01 [29]
Strength in shear directions (N/mm^2^)	3 [36]	0.64 [29]
Fracture energy (N/mm)	0.2 (empirical)	2 (empirical)

**Table 4:** Mesh size sensitivity analysis.

Model ID	Mesh Size (mm)		Computational Time (s)
	Beam	Cement	
Model 1	100	100	39
Model 2	50	50	208
Model 3	25	25	1456
Model 4	20	20	2925
Model 5	15	15	6699
Model 6	10	10	20,800

**Table 5:** Ultimate loads and corresponding deflection obtained from FE analyses.

Specimens	Ultimate Loads (kN)	Deflection at Ultimate (mm)	$\frac{F_{u}}{F_{ACI318}}$	$\frac{F_{u}}{F_{ACI549}}$	$\frac{F_{u}}{F_{ACI440}}$
C0	48.96	8.44	1.15	⸺	⸺
C10	33.02	6.36	1.26	⸺	⸺
C20	28.38	5.25	1.29	⸺	⸺
C30	24.40	4.10	1.38	⸺	⸺
C40	21.38	3.74	1.60	⸺	⸺
C0-Pt	56.33	12.80	⸺	1.24	1.31
C10-Pt	41.69	14.93	⸺	1.37	1.39
C20-Pt	37.24	13.08	⸺	1.37	1.40
C30-Pt	31.67	13.61	⸺	1.33	1.36
C40-Pt	28.87	12.86	⸺	1.41	1.44
C0-Pt-D10	56.18	13.18	⸺	1.24	1.30
C0-Pt-D20	55.96	13.37	⸺	1.23	1.30
C0-Pt-D30	55.53	10.90	⸺	1.23	1.29
C0-Pt-D30-Y	64.15	14.33	⸺	1.42	1.49
C0-Pt-D40	54.73	10.67	⸺	1.21	1.27
C0-Pt-D40-Y	63.02	13.59	⸺	1.39	1.46
Mean, Pm			1.34	1.31	1.36
COV, VP			0.114	0.060	0.054

## Data Availability

The data presented in this study are available on request from the corresponding author. The data are not publicly available because the research is still on going.
